# Single and Multitarget Systems for Drug Delivery and Detection: Up-to-Date Strategies for Brain Disorders

**DOI:** 10.3390/ph16121721

**Published:** 2023-12-12

**Authors:** Clara Grosso, Aurora Silva, Cristina Delerue-Matos, Maria Fátima Barroso

**Affiliations:** 1 REQUIMTE/LAQV, Instituto Superior de Engenharia do Porto, Instituto Politécnico do Porto, Rua Dr. António Bernardino de Almeida 431, 4249-015 Porto, Portugal; mass@isep.ipp.pt (A.S.); cmm@isep.ipp.pt (C.D.-M.); mfb@isep.ipp.pt (M.F.B.); 2 Nutrition and Bromatology Group, Analytical and Food Chemistry Department, Faculty of Food Science and Technology, Ourense Campus, Universidad de Vigo, E-32004 Ourense, Spain

**Keywords:** blood–brain barrier, nanoparticles, drug delivery, early diagnosis, multifactorial disorders

## Abstract

This review summarizes the recent findings on the development of different types of single and multitarget nanoparticles for disease detection and drug delivery to the brain, focusing on promising active principles encapsulated and nanoparticle surface modification and functionalization. Functionalized nanoparticles have emerged as promising tools for the diagnosis and treatment of brain disorders, offering a novel approach to addressing complex neurological challenges. They can act as drug delivery vehicles, transporting one or multiple therapeutic agents across the blood–brain barrier and precisely releasing them at the site of action. In diagnostics, functionalized nanoparticles can serve as highly sensitive contrast agents for imaging techniques such as magnetic resonance imaging and computed tomography scans. By attaching targeting ligands to the nanoparticles, they can selectively accumulate in the affected areas of the brain, enhancing the accuracy of disease detection. This enables early diagnosis and monitoring of conditions like Alzheimer’s or Parkinson’s diseases. While the field is still evolving, functionalized nanoparticles represent a promising path for advancing our ability to diagnose and treat brain disorders with greater precision, reduced invasiveness, and improved therapeutic outcomes.

## 1. Introduction

The prevalence of neurodegenerative and neuropsychiatric disorders is increasing dramatically. According to the World Health Organization (WHO), in 2023, more than 55 million people have dementia worldwide, with Alzheimer’s disease (AD) being the most common form (60 to 70% of cases) [1], while the data from 2019 indicate that the global prevalence of Parkinson’s disease (PD) was 8.5 million individuals [2]. The pathogenesis of AD is a complex process that remains unclear. The aggregation of beta-amyloid (Aβ) to form senile plaques and oligomers of Aβ, aggregation of tau to form neurofibrillary tangles (NFTs), change in acetylcholine levels, oxidative stress, and neuroinflammation are the main hallmarks in AD. However, AD is still managed with symptomatic treatments consisting of acetylcholinesterase inhibitors (AChEIs) (donepezil, rivastigmine, and galantamine) and/or N-methyl-D-aspartate (NMDA) receptor antagonists (memantine). Although being a topic of debate, Aducanumab, an Aβ-directed monoclonal antibody, was granted accelerated approval by the US Food and Drug Administration (FDA) in 2021 as the first disease-modifying therapy for AD. Other mechanisms and pathways of interest are being explored to develop new therapies, including focusing in other neurotransmitters, metabolism, the neuroimmune system, the microbiome, and the activity of a variety of nutraceuticals [3]. PD is characterized by the presence of Lewy bodies (LBs) and Lewy neurites (LNs), which are intraneuronal inclusion bodies observed in postmortem histopathological examinations of the brains of PD patients. The prodromal phase of PD is marked by a variety of non-motor symptoms that can precede the emergence of classical motor symptoms by several years. These early symptoms encompass issues such as constipation, gastric motility problems, sleep disruptions, depression, anxiety disorder, and cognitive impairments. As the disease progresses to stages 3 and 4, it affects key brain regions like the substantia nigra, thalamus, and amygdala, and the characteristic motor symptoms become clinically apparent. At stages 5 and 6, the pathology extends to sensory and motor areas in the neocortex, culminating in the most pronounced manifestation of the disease. In advanced stages of PD, patients may experience more severe motor symptoms, including instability, an increased risk of falls, difficulty swallowing (dysphagia), and non-motor symptoms like dementia and psychosis [3]. Currently, dopaminergic drugs are used to control the progression of the disease [4].

Exploring novel neuroprotective drugs with different mechanisms of action from those already used, combining drugs aiming at a multitarget approach, or finding new delivery routes to deliver the currently available drugs more efficiently to the brain are three possible ways to meet the requirements of the patients. In spite of new or combined mechanisms of action, multitarget therapy can offer multiple advantages, being more efficient than the conventional single-target treatment approaches. This can be achieved either using “cocktail drug–multiple targets” or “one compound–multiple targets” approaches [5]. For instance, combining α-tocopherol (an antioxidant) with donepezil (a cholinesterase inhibitor) showed promising results in an animal model of AD [6], as well as the co-delivery of BACE1 antisense sh-RNA and D-peptide, which induced both a reduction in Aβ plaque deposition and the inhibition of *p*-tau-related fibril formation in double transgenic (Tg) mice ((APPswe, PSEN1dE9)85Dbo/Mmjax), respectively [7]. Moreover, it has been shown that neuronal survival and repair processes were promoted by the combinatory effect of curcumin and docosahexaenoic acid [8]. Glinz et al. conducted a systematic review and meta-analysis of nine randomized controlled trials (2604 patients) comparing the clinical efficacy and safety of a combination therapy of AChEIsand memantine to monotherapy with either substance in patients with moderate to severe AD. The authors concluded that combined therapy had a significantly greater effect on cognition than AChEI monotherapy [9]. The benefits of combined therapy for AD were also highlighted by Kabir et al. [10]. Hybrid compounds have been designed for AD treatment by incorporating multiple known pharmacophores. For instance, compounds with dual-acting effects such as AChEI and butyrylcholinesterase inhibitor (BuChEI); AChEI and β-secretase (BACE 1) inhibitor; AChEI and glycogen synthase kinase 3 beta (GSK-3β) inhibitor; AChEI and NMDA antagonist; AChEI and serotonin receptor (5-HT_4_R) partial agonist; AChEI and serotonin transporter (SERT) inhibitor; ChEI and antioxidant; and AChEI and MAO-B inhibitor, among others, have been developed [5,11]. NLX-112, a non-dopaminergic drug (a highly selective 5-HT_1A_ receptor agonist), is a dual-effect drug, acting both on dyskinesia and the movement symptoms of PD [12].

Concerning neuropsychiatric disorders, depression and anxiety are the most common neuropsychiatric disorders, affecting approximately 280 [13] and 301 million people [14], respectively. Progress in discovering and developing new and improved psychiatric drugs has been slow and disappointing. The vast majority of currently prescribed drugs are arguably no more effective than the first generation of psychiatric drugs introduced in the 1950s and 1960s [15]. The etiology of depression (major depressive disorder) and anxiety are still not clear, but it is generally believed that they are multifactorial diseases caused by the interaction of social, psychological, and biological aspects. The symptoms include avoiding contact with friends; neglecting hobbies and interests; having difficulties in home, work, or family life; continuous low mood or sadness; feeling hopeless and helpless; having low self-esteem; feeling irritable and intolerant of others; having no motivation or interest in things; finding it hard to make decisions; moving or speaking more slowly than usual; changes in appetite or weight; unexplained aches and pains; and disturbed sleep. Despite several monoaminergic drugs being available on the market, 1/3 of depressive patients are unresponsive to this kind of treatment, Spravato^®^ (S-ketamine, an NMDA receptor antagonist) being their only hope [16,17]. However, concerns such as the transient adverse effects and potential misuse liability of Spravato^®^ (Janssen: Pharmaceuticals, Inc., Titusville, NJ, USA) have limited its widespread use [18]. Henssler et al. [19] performed a meta-analysis of 39 trials comprising 6751 patients and found that combined antidepressant therapy using a reuptake inhibitor with an antagonist of presynaptic α2-autoreceptors (mianserin, mirtazapine, trazodone) was associated with significantly superior treatment outcomes compared with antidepressant monotherapy. 

Symptoms of anxiety include feeling restless; being easily fatigued or irritable; having difficulty in concentrating; having unexplained pains (e.g., headaches, muscle aches, stomachaches); having difficulty in controlling feelings of worry; and having sleep problems. The first-line treatment for anxiety is based on selective serotonin reuptake inhibitors (SSRIs), serotonin–norepinephrine reuptake inhibitors (SNRIs), and benzodiazepines that target the gamma-amino butyric acid (GABA) receptor. However, the drugs currently in clinical development for the treatment of anxiety disorder include other mechanisms of action, namely NMDA receptor antagonism, negative allosteric modulation of metabotropic glutamate receptors 1–5, sodium channel inhibition, vasopressin receptor 1A antagonism/agonism, antagonism of orexin receptors 1,2, and agonism of neuropeptide Y receptors 1,2,5 [20,21].

**Figure 1:**
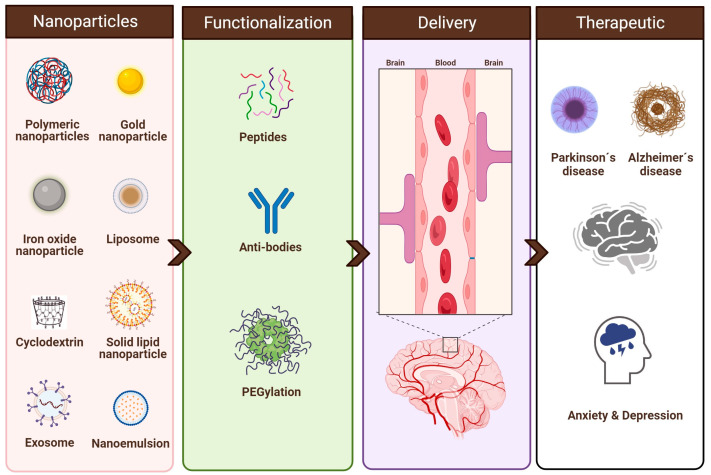
Most common types of nanoparticles and functionalization strategies for brain disorder diagnostics and treatment. Created using BioRender.com (accessed on 15 October 2023).

Other important aspect deals with the delivery route of drugs to the brain. Developing an efficient therapeutic system for brain disorders faces a significant challenge due to the presence of the blood–brain barrier (BBB), a barrier that separates the blood and the brain interstitial fluid and obstructs the passage of drugs, peptides, and large molecules into the brain [22]. Several strategies have been developed to increase the passage of the drugs through the BBB. Thus, this review paper provides a comprehensive analysis of the latest developments in nanoparticle-based drug delivery and diagnostics for brain disorders, encompassing neurodegenerative (AD and PD) and neuropsychiatric conditions (depression and anxiety). An array of nanoparticles, including lipid nanoparticles (liposomes, exosomes, solid lipid nanoparticles, and nanoemulsions), polymeric nanoparticles (PLGA, chitosan, alginate-based polymeric nanoparticles, and cyclodextrins), as well as magnetic nanoparticles, have emerged as promising tools in the quest to enhance the therapeutic and diagnostic outcomes for these challenging diseases. Strategies to functionalize these nanoparticles will also be discussed in the next sections (Figure 1). 

## 2. Materials and Methods

This review underscores the significance of nanoparticle-based drug delivery and diagnostic platforms in addressing the complexities of brain disorders. We offer a detailed and up-to-date survey of these developments published between 2010 and 2023, with a focus on their design, applications, and potential for improving the management of brain disorders. The keywords selected for the database search included: “liposome”, “exosome”, ”solid lipid nanoparticle”, “nanoemulsion”, “polymeric nanoparticles”, “PLGA”, “Chitosan”, “cyclodextrins”, “nanoparticle functionalization”, “covalent bonding”, “non-covalent bonding”, “magnetic nanoparticles”, “depression”, “major depressive disorder”, “anxiety”, Alzheimer’s disease”, “Parkinson’s disease”, “blood–brain barrier” “cytotoxicity”, and a combination of these terms. Thus, 93 studies were selected based on the following criteria: (1) the abstract and entire text available; (2) documents written in the English language; (3) contained information regarding nanoparticle composition, physicochemical characteristics, and pharmacological effects. 

## 3. Blood–Brain Barrier and Delivery Routes

**Figure 2:**
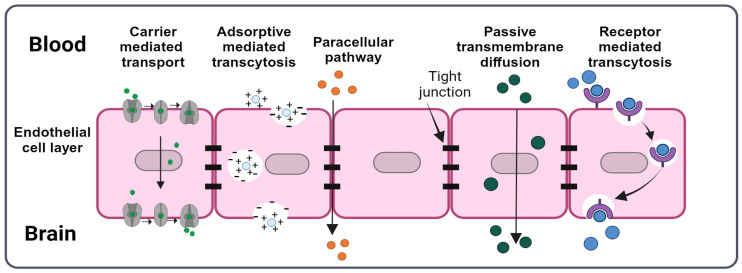
Transport mechanism across the BBB. Created using BioRender.com (accessed on 20 November 2023).

The BBB’s intricate structure comprises brain capillary endothelial cells, pericytes, perivascular mast cells, basement membranes, astrocytes, and neuronal cells, collectively responsible for regulating the exchange of molecules between the bloodstream and the brain. Of particular importance are the brain capillary endothelial cells, which play a crucial role in safeguarding the brain via the formation of tight junctions. These tight junctions serve as a primary defense mechanism by forming a high-resistance paracellular barrier limiting the crossing of molecules, ions, harmful toxins, and pathogens into the brain [22]. Transcellular transport, on the other hand, is regulated by specialized transporters, pumps, and receptors. Efflux transport systems, metabolizing enzymes, and multiple membrane transporters and receptors exist in the brain capillary endothelial cells, reinforcing the barrier properties of the BBB. They are responsible for removing undesirable substances from the brain into the systemic circulation [23,24]. Examples of these systems include multidrug resistance transporters, monocarboxylate transporters, and organic anion transporters [23]. Figure 2 exemplifies the five main transport mechanisms to transport drugs or nanocarriers across the BBB.

Passive transport occurs via transmembrane transport (transcellular) or tight junctions (paracellular). Only small lipophilic molecules, with a molecular weight < 500 Da and less than eight hydrogen bonds, or small gas molecules (such as CO_2_ or O_2_) can escape the P-glycoprotein (P-gp)-type multidrug resistance efflux pumps and freely diffuse through the BBB via transmembrane diffusion. However, the transport of most nutrients requires specialized transporters (carriers), receptors, or vesicular processes (such as adsorptive transcytosis). Carrier-mediated transport includes the use of large neutral amino acid transporters (LAT1) for the transport of amino acids, nucleosides, and certain drugs, and of the glucose transporter (GLUT1) for glucose transport, while several receptors are involved in receptor-mediated transport, such as transferrin receptor (TfR), insulin receptor (INSR), insulin-like growth factor-1 receptor (IGF1R), and leptin receptor (LEPR), among others. Positively charged molecules (e.g., polymers, cationic lipids, albumin, and nanoparticles) undergo adsorptive endocytosis, due to interactions with the negatively charged cell membrane. In paracellular transport, small water-soluble molecules pass through small intercellular pores in tight junctions, possibly involving claudins [22,23,25,26].

Different strategies have been suggested to deliver drugs to the brain: (a) drugs can be delivered to the brain via local delivery (injection via a catheter). Biodegradable polymer implants can also be used for sustained release of drugs. These procedures require surgery, are highly invasive, and are mostly used to treat glioblastomas or other brain tumors; (b) drugs can be delivered via the intranasal route. After reaching the nasal cavity, a drug loaded inside nanocarriers can be transported along the olfactory bulb (olfactory pathway) and the trigeminal nerve (trigeminal pathway) directly to the central nervous system (CNS), circumventing the BBB. Although an extremely attractive option, the intranasal route has drawbacks such as a high variability of the delivered dose depending on the state of the nasal mucosa; (c) the most popular delivery route remains the systemic pathway, in which drugs have to cross the BBB; (d) finally, an innovative way to solve the permeation problem with the BBB is to load drugs inside nanoparticles [27]. Within this context, the surface functionalization of nanoparticles is commonly employed as a strategy to enhance the drug’s ability to traverse the BBB, as well as to reduce toxicity and to increase nanoparticles stability in biological fluids [28]. Different methods for nanoparticle surface modification have been employed, namely chemical ligand exchange (which involves replacing existing surface ligands on nanoparticles with new ones) [29,30,31], covalent bonding (based on the attachment of ligands directly to the nanoparticle surface by sharing electrons) [32], and adsorption or non-covalent bonding (involving adherence of molecules onto the nanoparticle surface via weak forces, for instance, van der Waals, electrostatic or hydrogen bonding) [33,34]. Non-covalent bonding has the advantage of being relatively simple; however, covalent bonding is more robust and can combat detachment irrespective of the process used [28,35].

Among the most common ligands used to functionalize nanoparticle’s surface are antibodies (e.g., the single chain fragment of variable region 46.1 (scFv46.1) [36]; the OX26 monoclonal antibody to the rat transferrin receptor; and monoclonal antibodies to the insulin receptor [37]) RNA aptamers [38], transferrin (targeting the transferrin receptor at the BBB), lactoferrin (targeting the lactoferrin receptor at the BBB), glucose (targeting glucose transporters at the BBB), apolipoprotein E (ApoE) (targeting the low-density lipoprotein receptor at the BBB), glutathione (targeting the Na^+^-dependent GSH transporter at the BBB), and angiopep-2 (which has good transcytosis ability across the BBB) [22].

Nanoparticles can also be modified with polyethylene glycol (PEG), which acts as a shield to protect nanoparticles from plasma protein binding and prevents opsonization activity and phagocytosis [39]. This is extremely important since uncoated nanoparticles are recognized as foreign agentsbeing readily cleared from systemic circulation by the cells of the mononuclear phagocyte system (MPS), barring accumulation in target cells and tissues. The MPS is composed of dendritic cells, blood monocytes, granulocytes, and tissue-resident macrophages in the liver, spleen, and lymph nodes that are responsible for clearing, processing, and degrading exogenous agents in the bloodstream [40].

Dual- and multitarget nanoparticles represent advanced nanoplatforms that go beyond the conventional approach by incorporating not only the core scaffold but also two or more functionalizing ligands. This innovative design facilitates the precise delivery of therapeutic agents to their intended destinations [41]. For instance, Arora et al. [42] demonstrated an efficient brain-targeted delivery of ApoE2-encoding plasmid DNA (pApoE2) using liposomes. The liposomes were functionalized with a glucose transporter-1 targeting ligand mannose (MAN) and a cell-penetrating peptide, rabies virus glycoprotein peptide (RVG29), or penetratin (Pen). Dual (RVGMAN and PenMAN)-functionalized liposomes were developed, taking advantage of the high density of glucose transporter-1 in the luminal side of the BBB and that cell-penetrating peptides have the ability to cross cell membrane bilayers, enhancing the transport of different cargo across the BBB [42,43]. Indeed, experiments with an in vitro BBB model (coculture of primary astrocytes and bEnd.3 cells) showed that the dual-modified liposomes promoted ∼2 times more protein expression than other formulations (monofunctionalized and unmodified liposomes) in neurons cultured below the in vitro BBB model. Neves et al. [44] encapsulated curcumin in solid lipid nanoparticles and observed that functionalization with transferrin produced a 1.5-fold increase in the permeability across hCMEC/D3 cells compared with the non-functionalized nanoparticles. Similarly, resveratrol-loaded solid lipid nanoparticles functionalized with ApoE also crossed the endothelial monolayer more efficiently (1.8-fold higher) than the non-functionalized nanoparticles [45]. ApoE-modified solid lipid nanoparticles were found to be internalized by clathrin-mediated endocytosis [46]. Thiazolidinedione-loaded PLGA nanoparticles decorated with a monoclonal anti-transferrin receptor antibody (8D3 mAb) were synthetized by Monge et al. [47]. The authors also produced three other nanoparticles—bare PLGA nanoparticles, 8D3-PLGA nanoparticles, and PLGA–thiazolidinedione—and incubated them with human serum to simulate bloodstream conditions. LC-MS/MS analysis revealed different protein corona compositions among functionalized and non-functionalized nanoparticles, with the 8D3-PLGA-thiazolidinedione and 8D3-PLGA nanoparticles being the only types of nanoparticles containing afamin and with ApoE and ApoA-I adsorbed into their protein corona. Afamin may play an important role in regulating vitamin E uptake, favoring its transport across the BBB. Likewise, apolipoproteins are involved in promoting the transport of drugs across the BBB [47].

## 4. Types of Nanoparticles

### 4.1. Lipid-Based Nanoparticles 

Liposomes are the most explored nanocarriers used in drug delivery due to their biocompatibility, stability, ease to synthesize, high drug loading efficiency, and high bioavailability. Due to their combined hydrophobic and hydrophilic characteristics, both polar and apolar drugs can be encapsulated in the aqueous interior core or in the lipophilic membrane, respectively. They are spherical phospholipid-based vesicles (with diameters between 50 and 500 nm) composed of one or more lipid bilayers, being classified as small unilamellar vesicles (SUV), large unilamellar vesicles (LUV), multilamellar vesicle (MLV), and multivesicular vesicles (MVV). To enhance the stability of the liposomal structure and prevent the leakage of its inner cargo, cholesterol or sphingomyelin can be introduced into the lipid mixture. These additives effectively regulate the membrane permeability, alter fluidity, and enhance the robustness of bilayer membranes [48,49]. The phospholipids frequently used in liposome composition include natural lipids such as phosphatidylcholine, sphingomyelin, and lecithin or synthetic lipids like 1,2-dipalmitoyl-sn-glycero-3-phosphocholine and ethyl-phosphatidylcholine [50]. 

Several liposome formulations have been developed to incorporate neuroprotective drugs, aiming at treating neurodegenerative and neuropsychiatric disorders (Table 1). Moreover, liposomes are also used for diagnostic purposes. Congo red can bind with amyloid plaques, and liposomes modified with Congo red have been patented for AD diagnosis [49]. 

Exosomes are a class of extracellular biovesicles of endocytic origin, which are constitutively or inductively released by virtually all cell types and are accessible in almost all body fluids, such as saliva, urine, plasma, cerebrospinal fluid, amniotic fluid, and semen [51]. These nanostructures originate from budding processes occurring upon the fusion of multivesicular bodies and the plasma membrane and are involved in many biological functions, especially communication and transfer of biological information between cells [51]. They are involved in synaptic plasticity, neuroinflammation, neurotoxicity, neurotransmission, neuronal stress, neurodevelopment, neurogenesis, and neurodegeneration in the brain [52]. 

**Table 1:** Examples of drug loaded into liposomes for neurodegenerative and neuropsychiatric disorder treatment.

Target/Disease	Drug(s)	Nanoparticle Type	Composition	Functionalization	Diameter of the Optimized Formulation(s) (nm)	Observations	Ref.
AD	Anti-amyloid single-domain antibody fragment (VHH-pa2H)	Liposome	1,2-dimyristoyl-sn-glycero-3-phosphocholine or egg-yolk phosphatidylcholine	Glutathione-DSPE-PEG2000 (PEG)	108, 110	Liposomes were injected into APPswe/PS1dE9 double transgenic mice, a mouse model of AD. GSH-PEG liposomes increased VHH-pa2H uptake in the brain.	[53]
AD	ApoE2-encoding plasmid DNA/chitosan	Liposome	Dioleoyl-3-trimethylammonium-propane chloride (DOTAP), dioleoyl-sn-glycero-3phosphoethanolamine (DOPE)	DSPE-PEG2000, Glucose transporter-1 (Glut-1) targeting ligand mannose (MAN)+ cell penetrating peptides (CPP) (rabies virus glycoprotein peptide (RVG) or penetratin (Pen))	168, 172	Significantly higher expression of ApoE2 in bEnd.3 cells, primary neurons, and astrocytes compared to monofunctionalized and unmodified liposomes. Dual-modified liposomes (RVG-MAN and Pen-MAN) also showed ∼2 times higher protein expression than single-targeted formulation (MAN or Pen) in neurons cultured below an in vitro BBB model.	[42]
AD	Rivastigmine	Liposome	Cholesterol and dipalmitoylphosphatidyl choline (DPPC) methylcellulose dimethyl-β-cyclodextrin or sodium taurocholate	Absorption enhancers (dimethyl-β-cyclodextrin or sodium taurocholate)	3.4 *, 4.8 *	Rivastigmine liposomes and solutions were also administered to mice orally and intraperitoneally. The highest AChE inhibition was observed for rivastigmine-sodium-taurocholate liposomes.	[54]
AD	Rivastigmine	Liposome	Egg phosphatidylcholine, cholesterol	DSPE-PEG2000-CPP	179	Intranasal administration of rivastigmine liposomes to rats demonstrated the capacity to improve rivastigmine distribution and adequate retention in CNS regions (hippocampus and cortex) and affected acetylcholinesterase (AChE) and butyrylcholinesterase (BuChE) activities. The effects were more pronounced that those induced by free rivastigmine and uncoated liposomes.	[55]
AD	Rivastigmine	Liposome	1,2-diacyl-sn-glycero-3-phosphocholine, dihexadecyl phosphate, cholesterol	-	68, 529	Liposomes resulted in faster memory regain and the amelioration of metabolic disturbances in AlCl_3_-treated rats than free rivastigmine solution.	[56]
AD	α-tocopherol + donepezil hydrochloride	Liposome	L-α-phosphatidylcholine and cholesterol	Tetradecyltriphenylphosphonium bromide	105–115	Intranasal administration to APP/PS1 mice resulted in enhanced learning abilities and a reduction in the formation rate of amyloid beta (Aβ) plaques in the entorhinal cortex and hippocampus of the brain.	[6]
AD	Galantamine hydrobromide	Liposome	Soya phosphatidylcholine and cholesterol	-	112	Intranasal administration of the liposomes could readily transport galantamine into rat brain tissues where it could inhibit cholinesterase.	[57]
AD	Quercetin	Liposome	Egg L-α-phosphatidylcholine, cholesterol	-	-	Effect of nasal administration of quercetin liposomes on neurodegeneration in an animal model of AD. Quercetin liposomes attenuated the degeneration of neurons and cholinergic neurons in the hippocampus, promoted the elevation of superoxide dismutase, catalase, and glutathione peroxidase activities and induced the reduction in malondialdehyde in the hippocampus.	[58]
PD	Levodopa	Liposome	Hydrogenated soybean phosphatidylcholine, cholesterol	DSPE-PEG2000-Chlorotoxin	107	After intraperitoneal injection to mice, liposomes loaded with levodopa significantly increased the distribution of dopamine (DA) and dihydroxyphenyl acetic acid in the substantia nigra and striata. In a 1-methyl-4-phenyl-1,2,3,6-tetrahydropyridine (MPTP)-induced PD mice model, levodopa-loaded chlorotoxin liposomes significantly attenuated the serious behavioral disorders and diminished the MPTP-induced loss of tyrosine hydroxylase-positive dopaminergic neurons.	[59]
PD	Glial-cell-derived neurotrophic factor (GDNF)	Liposome	Dioleoylphosphatidylcholine (DOPC), cholesterol, and stearylamine	-	149	Rats were nasally administered with GDNF solution or with cationic liposomes containing GDNF before the injection of 6-hydroxydopamine (6-OHDA). Both intranasal GDNF treatments induced a neurotrophic effect in the substantia nigra since the number of tyrosine hydroxylase (TH)-positive neurons was significantly higher than in controls given intranasal PBS liposomes.	[60]
PD	Dopamine	Liposome	Cholesterol, 1,2-dioleyl-sn-glycero-3-phosphocholine (DOPC), L-α-phosphatidic acid	APP-derived peptide	100	Intraperitoneal injection of the APP-targeted liposomes loaded with dopamine resulted in a significant increase in striatal DA in amphetamine-treated mice.	[61]
PD	Resveratrol	Liposome	Soybean lecithin and cholesterol	-	146–585	Resveratrol liposomes could significantly enhance the activity of mitochondrial electron transfer chain complex I in the substantia nigra cells of 6-hydroxydopamine-treated rats, promote the expression of complex I subcomponent MT-ND1-37kD, improve mitochondrial membrane potential, inhibit the release of mitochondrial cytochrome C and apoptotic inducible factor, enhance the expression of mitochondrial functional protein PINK1, increase the phosphorylated TRAP1 level, and elevate the phosphorylated TRAP1/TRAP1 levels.	[62]
PD	Polyphenol-rich grape pomace extracts	Liposome	Brain lipids, 1,2-distearoylsn-glycero-3-phosphoethanolamine-N-(PEG)5000	anti-transferrin receptor antibody	133	The antioxidant nanoplatform was successfully tested in a rotenone-induced in vitro PD model, where it completely regulated the reactive oxygen species (ROS) levels, prevented the aggregation of α-synuclein fibrils, and restored cell viability.	[63]
Anxiety	Nimodipine	Liposome	Soybean phosphatidylcholine and cholesterol	-	107	The results suggest that the administration of nimodipine liposomes has no sedative or muscle relaxant effect in animals but displayed anxiolytic-like activity in bright-field test. The results obtained in the open arms test suggest that the new formulation acts on benzodiazepine receptors.	[64]
Anxiety	Eugenol	Liposome	Lecithin, cholesterol	-	91	The mRNA expression of glyoxylase-1 (GLO-1) and GLO-1 protein expression were measured in 42 BALB/c mice submitted to stress using a conventional restraint model. The mRNA and protein expressions were found to be increased in animals given anxiety as compared to the normal control. Eugenol and its liposome-based nanocarriers counteracted this behavior, with liposomal eugenol behaving better than the compound alone.	[65]
Anxiety	Eugenol	Liposome	Lecithin, cholesterol	-	91	Stress was induced in 42 BALB/c mice using a conventional restraint model. The mRNA expression of neurokinin 1 receptor (NK1R) and the NK1R protein expression were increased in animals given anxiety as compared to the normal control. Both parameters decreased in animals treated with eugenol and its liposome-based nanocarriers, the results being better for nanocarriers.	[66]
Anxiety/Depression	Quercetin	Liposome	Egg L-α phosphatidylcholine, cholesterol	-	-	Wistar rats were intranasally administered quercetin liposomes. These nanocarriers possessed anxiolytic and anti-depression like activity and a cognitive-enhancing effect, which were assessed using the elevated plus maze test, forced swimming test, and Morris water maze test	[67]
Depression	Plasmid-harboring brain-derived neurotrophic factor (BDNF)	Liposome	1,2-Dioleoyl-sn-glycero-3-phosphoethanolamine (DOPE), and 1,2-dioleoyl-3-trimethylammonium-propane chloride (DOTAP), cholesterol	DSPE-PEG2000, transferrin, and arginine	125	Physicochemical characterization of the nanoparticles produced.	[68]
Depression	Nimodipine	Liposome	Soybean phosphatidylcholine and cholesterol	-	107	Tail suspension test, forced swimming test, and MAO-B activity assay suggested that nimodipine-liposomes displayed more antidepressant activity than imipramine and paroxetine but with a lower effect than that observed in the group receiving liposomes + reserpine.	[69]
Depression	Sertraline hydrochloride	Liposome	Hydrogenated soya phosphatidylcholine-L-α-phosphatidylcholine (HSPC), distearoyl phosphatidyl glycerol sodium (DSPG) and cholesterol	-	152	The use of liposomes increased drug transport to the brain compared with administration with free sertraline	[70]
Depression	Piperine	Liposome	Egg L-α-phosphatidylcholine, cholesterol	Polyethylene glycol 1000	100	Piperine-encapsulated liposomes displayed antidepressant-like activity in Wistar rats submitted to forced swimming, Morris water maze, and spontaneous motor behavior tests.	[71]

* μm. Alzheimer’s disease (AD), amyloid beta (Aβ), amyloid precursor protein (APP), mouse model of Alzheimer’s disease (APP/PS1), acetylcholinesterase (AChE), beta-secretase (BACE1), isogenic mice (BALB), blood–brain barrier (BBB), bovine serum albumin (BSA), brain-derived neurotrophic factor (BDNF), butyrylcholinesterase (BuChE), cell-penetrating peptide (CPP), dopamine (DA), dioleoyl-3-trimethylammonium-propane chloride (DOTAP), dioleoyl phosphatidylcholine or 1,2-dioleyl-sn-glycero-3-phosphocholine (DOPC), L-α-phosphatidic acid (DOPC), central nervous system (CNS), dioleoyl-3-trimethylammonium-propane chloride (DOTAP), dioleoyl-sn-glycero-3phosphoethanolamine (DOPE), dipalmitoyl phosphatidyl choline (DPPC), 1,2-distearoyl-sn-glycero-3-phosphoethanolamine-N-[amino(polyethylene glycol)-2000] (ammonium salt) (DSPE-PEG2000), epigallocatechin-3-O-gallate (EGCG), glial cell derived neurotrophic factor (GDNF), glutathione PEGylated liposomes (GSH-PEG), glyoxylase-1(GLO-1), 6-hydroxydopamine (6-OHDA), glucose transporter-1 targeting ligand mannose (MAN), monoamine oxidase (MAO), 1-methyl-4-phenyl-1,2,3,6-tetrahydropyridine (MPTP), mitochondrially encoded NADH dehydrogenase (MT-ND1), neurofibrillary tangles (NFTs), neurokinin 1 receptor (NK1R), Parkinson’s disease (PD), penetratin (Pen), poly ethylene glycol (PEG), PTEN-induced kinase-1 (PINK1), poly(lactic-co-glycolic acid) (PLGA), rabies virus glycoprotein peptide (RVG), reactive oxygen species (ROS), necrosis factor receptor-associated protein (TRAP), anti-amyloid single-domain antibody fragment (VHH-pa2H).

Exosomes are characterized by a single lipid bilayer membrane, containing phospholipids such as phosphatidylcholine, phosphatidylserine, phosphatidylethanolamine, phosphatidylinositol, phosphatidic acid, as well as cholesterol, ceramides, sphingomyelin, and glycine [51]. They also carry cell-specific cargo of proteins, lipids, and genetic materials (RNA and DNA), and can be selectively taken up by neighboring or remote cells, reprogramming the recipient cells [51,72,73].

Exosomes from various sources have revealed a wide array of constituent molecules, comprising approximately 4400 proteins, 194 lipids, 1639 mRNAs, and 764 miRNAs. These findings underscore the intricate nature of exosomes and hint at their potential for diverse functional roles [51,72]. Typically, exosomes are highly enriched in proteins with various functions, such as tetraspanins, which take part in cell penetration, invasion, and fusion events; heat shock proteins, as part of the stress response, which are involved in antigen binding and presentation; multivesicular body formation proteins that are involved in exosome release; and proteins responsible for membrane transport and fusion; metabolic enzymes; growth factors; and cytokines [51,72] Spherical and with diameters in the range of 40 to 150 nm, exosomes have garnered significant attention in targeted drug delivery research, primarily owing to their persistent presence within the circulatory system and their remarkable ability to shield their cargo from degradation by various proteases and nucleases. Compared to liposomes, exosomes have higher penetration, prolonged blood circulation, biocompatibility, and enhanced biodistribution [73]. 

Besides being a promising therapeutic vehicle, exosomes can also act as valuable biomarkers in diagnosing brain diseases. Within the CNS, exosomes play pivotal roles in various physiological processes, including intercellular communication, the maintenance of myelination, synaptic plasticity, antigen presentation, and providing trophic support to neurons. Nonetheless, in pathological circumstances, especially in conditions marked by abnormal protein accumulation, exosomes are increasingly involved in the removal of accumulated, undesirable biomolecules. Specifically, the disposal of unwanted cellular components via exosomes becomes a crucial mechanism when other cellular clearance systems, such as the proteasome and autophagy–lysosome system, gradually fail to effectively eliminate the aggregated proteins. In such situations, exosomes originating from the CNS have been observed to participate in cell-to-cell spreading of amyloidogenic proteins, namely Aβ, tau, and α-synuclein [74]. Aβ42, pT181-tau, pS396-tau, total-tau, and α-synuclein are some of the potential fluid biomarkers for the diagnosis of neurodegenerative diseases [74]. Moreover, there are distinct profiles of exosomal miRNAs even before the onset of irreversible neurological damage, indicating that exosomal miRNAs within tissues and biological fluids could serve as better biomarkers than aggregated proteins. Even more importantly, exosomes are highly enriched with miRNAs, compared to their parental cells and cell-free blood, making them of choice par excellence for biomarker profiling [75].

Solid lipid nanoparticles (SLN) are lipid-based biocompatible nanocarrier systems composed by lipids or modified lipids (e.g., triglycerides, fatty acids, or waxes) with a size diameter in the range of 10–1000 nm. These nanoparticles have a solid hydrophobic lipid core, in which both hydrophilic and lipophilic drugs can be dispersed. Compared to liposomes and polymeric nanoparticles, SLNs safeguard better the encapsulated drug from biochemical degradation due to the solid lipid nature of their core instead of the water-soluble core of other types of nanoparticles [76]. 

Examples of drug-loaded exosomes and SLNs containing bioactive compounds for brain disorder treatments are depicted in Table 2 and Table 3, respectively.

Nanoemulsions are colloidal biphasic dispersions systems consisting of two immiscible liquids: one is the dispersed phase and the other is a continuous phase that is stabilized using appropriate emulsifying agents (e.g., surfactant and co-surfactant). They can be of water-in-oil (w/o) or oil-in-water (o/w) types and their droplet or globular dimension typically varies from 200 nm to 100 µm. Nanoemulsions offer a wide range of applications since they are well absorbed through the mucosal layer and are hence suitable for site-specific drug delivery via different routes, namely the oral, parental, nasal, and ocular routes [94,95]. The higher encapsulation of lipophilic drugs in o/w-type nanoemulsions offers greater solubility, better absorption, and higher bioavailability with minimum enzymatic degradation, being the preferable type of nanoemulsion to permeate the BBB [95].

Nanoemulsions normally contain 5–20% oil/lipid droplets in the case of o/w emulsions. However, this percentage can be higher (up to 70%) [96]. Concerning the lipidic phase, several oils are employed, namely cottonseed oil, coconut oil, sesame oil, safflower oil, rice bran oil, and soybean oil. The use of fatty acids such as linolenic acid, omega-6 fatty acids, oleic acid, and linolenic acids alone or in combination has shown benefits for the development of nanoemulsions to be delivered via the intranasal route [95,96]. Surfactants like phosphatidylcholine, bilesalt, chitosan, starch, Tweens (Tween 60, 80), sodium dodecyl sulphate, cetyl pyridinium chloride, labrasol, Span (Span 60, 80), poloxamers, casein, PEG-containing block polymers, and certain proteins and lipids are commonly used to reduce the interfacial tension between both phases of the biphasic system and also to increase their stability. In addition, chiral alcohols, Tween 80, PEG 600, glycerol, butan-1-ol, transcutol-P, and sorbitol are included in the formulations as co-surfactants [95,96]. Preservatives, antioxidants, and chemoprotectants are also included in nanoemulsion composition. Among them, benzoic acid, sorbic acid, propionic acid, and dehydro-acetic acid have antifungal properties, and ascorbic acid, citric acid, tocopherols, butylated hydroxytoluene (BHT), and butylated hydroxyanisole (BHA), among others, are antioxidant agents [96]. Table 4 resumes the application of drugs loaded into nanoemulsions for neurodegenerative and neuropsychiatric disorder treatment.

### 4.2. Polymeric Nanoparticles

Polymeric nanoparticles, ranging in size from 1 to 1000 nm, serve as versatile carriers for active compounds, which can either be encapsulated within or adsorbed onto their polymeric structure. Within this category, two distinct nanocarriers emerge, namely nanocapsules and nanospheres. Nanocapsules are composed of an oily core in which the drug is usually dissolved, enveloped by a polymeric shell responsible for regulating the controlled release of the drug from the core. Nanospheres are characterized by a continuous polymeric network that can either retain the drug within its matrix or adsorb it onto the nanoparticle’s surface [109].

Natural and synthetic polymers are usually used to prepare polymeric nanoparticles. Natural polymers include chitosan/chitosan derivatives, alginate, gelatin, and albumin, while polylactic acid (PLA), poly(lactic-co-glycolic acid) (PLGA), poly-ε-caprolactone (PLC), and poly(amido amine) are among the synthetic ones [110].

Another class of polymeric nanoparticles are cyclodextrins. Cyclodextrins (CDs) are a family of cyclic oligosaccharides produced from starch or starch-based materials by the bacterial enzyme cyclodextrin glycosyltransferase (CGT). CGTs from *Bacillus* species are the most extensively studied enzymes CDs, consisting of six (αCD), seven (βCD), eight (γCD), or even more glucopyranose monomers connected via α-1,4-glycosidic bonds. Cyclodextrins exhibit a unique structural geometry characterized by a truncated cone shape, featuring a hydrophilic outer surface and a more lipophilic interior cavity. Due to this configuration, cyclodextrins possess the remarkable property of being water-soluble while also being capable of accommodating hydrophobic molecules simultaneously. The inclusion of lipophilic substances into CDs enhances their stability and bioavailability while also increasing their aqueous solubility [111] The OH groups of CDs are also involved in the binding processes via electrostatic forces and Van der Waals and hydrogen bonding interactions [112]. There are several cyclodextrin-based drugs for brain disorders already on the market, for instance, Geodon^®^ IM, Pfizer Inc., New York, NY, USA (Captisol^®^-enabled ziprasidone mesylate for schizophrenia and bipolar disorder), Abilify^®^, Bristol-Myers Squibb, Princeton, NY, USA (Captisol^®^-enabled aripiprazole for bipolar I disorder), and Zulresso^®^, Sage Therapeutics, Inc., Cambridge, MA, USA (Captisol^®^-enabled brexanolone for moderate to severe postpartum depression) [113].

Multiple ligands can be affixed to the surface of polymeric nanoparticles to aid in their transport through the BBB. There are four primary categories of ligands employed for the functionalization of polymeric nanoparticles. These include ligands that adsorb proteins from the bloodstream, enabling them to interact directly with receptors or transporters found on the endothelial layer of the BBB (e.g., Tween 80); ligands that directly bind to BBB receptors or transporters (e.g., antibodies designed for receptors like the transferrin receptor, insulin receptor, or glucose transporter); ligands that enhance the charge and hydrophobicity of the nanoparticles, such as amphiphilic peptides; and ligands that extend the circulation time of nanoparticles in the bloodstream, such as PEG [114].

Table 5 presents some examples of neuroprotective drugs encapsulated in polymeric nanoparticles.

### 4.3. Metallic Nanoparticles

Metallic nanoparticles for biomedical purposes include gold nanoparticles, magnetic nanoparticles, and silver nanoparticles. Materials such as pure metals (Fe, Co, and Ni), alloys (FeCo, Alnico, and Permalloy), and ferrites (Mn_0.6_Zn_0.4_Fe_2_O_4_ and CoFe_2_O_4_) with high saturation magnetizations are usually selected to produce magnetic nanoparticles for biomedical applications, namely, magnetic hyperthermia (for the focal treatment of tumors), contrast agents for magnetic resonance imaging (MRI), and targeted drug delivery systems. Because of the oxidative characteristics and high toxicity levels of pure metals, they are not suitable for in vivo use. Iron oxides (e.g., Fe_3_O_4_, γ-Fe_2_O_3_) are highly stable and biocompatible [138,139].

With exceptional spatial resolution and tissue contrast, MRI offers detailed anatomical images of soft tissues, establishing itself as a pivotal diagnostic modality for imaging the brain, cartilage, heart, and blood vessels, and tumor detection. In contrast to other imaging platforms like computerized axial tomography (CAT), positron emission tomography (PET), and single-photon emission computed tomography (SPECT), MRI techniques eliminate the need for radioactive agents and ionizing radiation. Despite its ability to generate detailed soft tissue images, MRI’s intrinsic low sensitivity poses challenges in distinguishing normal tissues from lesions. The introduction of contrast agents addresses this limitation by amplifying the contrast effect in regions of interest via the acceleration of magnetic relaxation [140]. Although more expensive than analogic MRI systems, modern digital MRI systems play a crucial role in acquiring, processing, and displaying the images produced during the scanning process. Moreover, digitalization has had a significant impact on healthcare in recent years, mainly in telemedicine. It can be principally useful for people in rural or underserved areas who may not have easy access to healthcare facilities [141]. 

Several authors have produced iron oxide nanoparticles (IONPs) for MRI purposes [142,143,144,145,146,147]. For instance, Tang et al. [147] have developed sulfated dextran-coated iron oxide nanoparticles for the imaging of activated microglia during brain inflammation. However, uncoated IONPs usually have some degree of toxicity, inducing, at high concentrations, increased generation of reactive oxygen species, reduction in cellular proliferation, or even induction of cell death [145]. As shown by Yu et al. [148], uncoated superparamagnetic iron oxide particles (SIONPs, 0.5 mg/mL) displayed up to a 6-fold increase in cytotoxicity compared to dextran-coated or PEG-coated iron oxide nanoparticles. Yang et al. [144] evaluated the potential reproductive toxicity of IONPs in ICR mice. The results showed that the nanoparticles could cause reversible damage to the reproductive system of male mice without affecting the main organs.

Various ligands specific to Aβ plaques have been found and utilized to functionalize or be encapsulated in nanoparticles to achieve early detection and treatment for AD. For instance, Cheng et al. [149] functionalized SIONPs loading curcumin, a natural compound that binds to Aβ plaques, and successfully visualized them in transgenic mouse brains using MRI. Near Aβ plaques, there is a deposition and accumulation of ferritin protein. Fe_3_O_4_-dextran coated magnetic nanoparticles were conjugated with anti-ferritin antibody to be developed as contrast agents to detect Aβ plaques. In both in vitro and in vivo tests (intravenous injection), the nanoparticles were able to recognize and bind specifically to the ferritin protein accumulated in the subiculum area of the AD transgenic mice [150]. 

Kim and Chang [151] investigated the therapeutic potential of human adipose-derived stem cells (hASCs) using magnetic nanoparticles in a 6-OHDA-induced PD mouse model. MRI showed hASC distribution in the substantia nigra of hASC-injected PD mice and in behavioral evaluations (apomorphine-induced rotation and Rota rod performance), hASCs significantly induced recovery in 6-OHDA-treated PD mice when compared with a saline-treated control group.

Magnetic, silver, and gold nanoparticles have also been tested for neurodegenerative and neuropsychiatric disorder treatment. For instance, Sivaji and Kannan developed gold nanoparticles conjugated with donepezil and functionalized them with polysorbate 80 and polyethylene glycol [152]. This nanocarrier was able to cross the zebrafish BBB and showed an increase in AChE inhibition activity of 30–38% in the zebrafish brain. Silver nanoparticles containing an extract from *Mucuna pruriens* seeds (rich in L-DOPA) significantly lowered catalepsy symptoms in mice, showing its potential for PD treatment [153]. Kim et al. [154] compared the neuroprotective effect of anthocyanins alone and anthocyanin-loaded PEG/gold nanoparticles in Aβ1-42-injected mouse and in in vitro models of AD. Both anthocyanins alone or conjugated reduced the Aβ1-42-induced neuroinflammatory and neuro-apoptotic markers via inhibiting the p-JNK/NF-κB/p-GSK3β pathway in both in vivo and in vitro AD models, although the conjugated form was more efficient. 

Khadrawy et al. [155] evaluated the antidepressant effect of curcumin-coated iron oxide nanoparticles in a rat model of depression. Wistar male rats were intraperitoneally injected with reserpine to produce depressive symptoms. In depressed rats, the FST showed an increased immobilization time and a reduced swimming time, which were counterbalanced by curcumin-coated iron oxide nanoparticles. While depressed rats showed significantly decreased levels of serotonin, noradrenaline, DA, and glutathione and significantly increased levels of MDA, nitric oxide, glutathione S-transferases, monoamine oxidase A, AChE, Na^+^, K^+^, and ATPase activities in the cortex and hippocampus, treatment with nanoparticles for two weeks restored the levels of all these biomarkers to the control levels. 

## 5. Comparison between Different Types of Nanoparticles for Brain Delivery

This review summarized the latest advances in the encapsulation of drugs to be delivered to the brain. Different types of nanocarriers were considered, including lipid-based, polymeric, and metallic nanoparticles. The advantages and disadvantages of each type of nanoparticle are discussed below.

Concerning lipid-based nanocarriers, solid lipid nanoparticles offer numerous advantages, such as facile manufacturing, enhanced pharmaceutical stability, increased drug content, efficient drug release, and prolonged stability. Solid lipid nanoparticles are effective in encapsulating poorly water-soluble compounds owing to their elevated lipid content. In contrast, although liposomes are the most widely used lipid-based encapsulation nanocarrier, they face challenges in achieving high drug loading for hydrophobic drugs due to their limited bilayer space [156]. Nanoemulsions, on the other hand, boast easy preparation without the necessity for organic solvents or cosolvents. Primarily derived from edible oils, they entail a low risk of toxicity [156]. van der Koog et al. [157] recently reviewed the advantages and limitations of exosomes and liposomes. According to the authors, both vesicle types have their own advantages. While liposomes offer extensive control over contents, exosomes have the advantage of inherent biocompatibility and a complex biological composition that will be hard to fully recapitulate in liposomes. Moreover, exosomes are of natural origin, while liposomes are of synthetic origin. 

Akel et al. [158] compared the efficiency of PLGA nanoparticles and chitosan-modified solid lipid nanoparticles for the intranasal administration of insulin. Chitosan-coated solid lipid nanoparticles were the best nanocarriers since they increased the mucoadhesion, nasal diffusion, and drug release rate. Nonetheless, compared with solid lipid nanoparticles and liposomes, polymeric nanoparticles have some limitations, such as possible self-aggregation, which, when it occurs, can impact brain delivery [159].

Ross et al. [160] compared the stability of Au, Ag, Fe_2_O_3_, TiO_2_, and ZnO nanoparticles of 5, 20, and 50 nm in sterile-filtered water; cell culture media DMEM supplemented with 10% fetal bovine serum, 1% l-glutamine, 1% non-essential amino acids, and 1% penicillin-streptomycin; and human serum, to understand what would occur after intravenous delivery. The authors concluded that the gold and silver nanoparticles were the most stable, and, concerning the particle size, the 20 nm nanoparticles appeared to be the least stable size across materials.

## 6. Emerging Trends in Nanomedicine: Challenges and Future Directions

Managing and treating neurodegenerative and neuropsychiatric disorders present three main challenges. These challenges encompass: (1) the absence of advanced diagnostic tools for early disease detection, and in this sense, nanoparticles could be used for the construction of high-performance biosensors for detecting biomarkers at the early stages of diagnosis, either as amplifier labels for signal augmentation or as a practical option for biorecognition element immobilization. Moreover, nanoparticle-mediated clinical imaging technologies can provide observation of localized protein accumulation, playing a complementary role in CNS disease diagnosis; (2) the limitation of drugs in effectively crossing the BBB, necessitating the development of encapsulation strategies, as reviewed in the preceding sections; and (3) the deficiency in personalized disease management for timely decision-making in therapy [161,162]. The intersection between personalized medicine and nanomedicine occurs in both the diagnostic and therapeutic areas. At the diagnostic level, besides being used for exploring the status of specific drug targets, nanotechnology is also very useful for pharmacogenetic approaches and for in vitro and in vivo testing. In the therapeutic area, nanomedicine can tailor drugs based on individualized patient conditions [163].

Challenges related to safety, efficacy, and regulatory considerations pose significant hurdles in advancing personalized nanomedicine for CNS diseases toward clinical applications. More research needs to be conducted to gather more information about the pharmacokinetic profile of nanoparticles, tissue distribution, cytotoxicity, and side effects in order for regulatory agencies, like the FDA and EMA, to approve new nanoparticle-based therapies or diagnostic tools. Although some liposomes and metallic nanoparticles have gained regulatory approval for clinical use for cancer, for pain management, or as antifungal treatment [164], Sinerem^®^ (GUERBET, Paris, France), an ultrasmall super paramagnetic iron oxide (USPIO) contrast agent for MRI, was withdrawn from the market in 2008 by the EMA due to concerns raised in clinical trials [164,165]. As far as what concerns CNS disorders, only a few clinical trials have yet been conducted. One single-center open-label pilot study (NCT03815916) was developed between 2019 and 2021 and tested the efficacy of gold nanocrystals (CNM-Au8) in PD, and a phase-II trial (NCT03806478) will evaluate (between June 2023 and December of 2024) the efficacy of the intranasal delivery of APH-1105 for the treatment of mild to moderate AD in adults [166].

Another important aspect is related to nanoparticle production. While it is easy to obtain stable nanoparticles at a small scale, difficulties with the scaling up and stability of large-scale manufacture have been widely experienced. Indeed, increasing the installation size from the laboratory scale to the industrial scale presents many difficulties, such as the maintenance of high reproducibility, homogeneity, and control over properties [167].

Altogether, nanotechnology emerges as an essential avenue for implementing a personalized medicine approach; however, it confronts challenges, including mainly safety concerns and scalability issues.

## 7. Conclusions

Significant research efforts have been dedicated to uncovering innovative strategies for delivering neuroprotective drugs to the brain. Circumventing the BBB to achieve high drug concentrations within the brain is highly challenging, and nanotechnology is one of the most efficient approaches to this challenge. In this review, we compiled recent studies on the development of nanoparticles for brain drug delivery and diagnostics, including lipidic, polymeric, and metallic nanoparticles. Examples of nanoparticle-loaded natural and synthetic drugs for neurodegenerative and neuropsychiatric treatment were presented alongside magnetic nanoparticles for brain imaging. Nanoparticle functionalization strategies to promote active transport through the BBB were also discussed, including with antibodies, RNA aptamers, transferrin, lactoferrin, glucose, ApoE, glutathione, and angiopep-2. In spite of the extensive progress made in the field of nanotechnology to boost diagnostic and therapeutic strategies for neurological and neuropsychiatric disorders, the integration of nanomedicine into clinical studies remains an ongoing challenge. 

## Figures and Tables

**Table 2:** Examples of drugs loaded into exosomes for neurodegenerative disorder treatment.

Target/Disease	Drug(s)	Nanoparticle Type	Composition	Functionalization	Diameter of the Optimized Formulation(s) (nm)	Observations	Ref.
AD	Curcumin	Exosome	Macrophage RAW264.7 cells treated with curcumin	Exosome inherited the lymphocyte function-associated antigen 1 from their parental cells, a protein that interacts with the endothelial intercellular adhesion molecule 1	117	Exosome improved the solubility and bioavailability of curcumin and increased drug penetration across the BBB. Exosomes derived from curcumin-treated RAW264.7 cells relieved the symptoms of AD by inhibiting phosphorylation of the tau protein via activating the AKT/GSK-3β pathway.	[77]
AD	Quercetin	Exosome	Isolated from whole blood of SD rats	Inherited heat shock protein 70	150	The bioavailability of quercetin was enhanced via loading into the exosome, relieving the symptoms of AD in okadaic-acid-induced AD mice, by inhibiting the cyclin-dependent-kinase-5-mediated phosphorylation of tau and reducing the formation of insoluble neurofibrillary tangles (NFTs).	[78]
AD	GAPDH small interfering RNA	Exosome	Bone marrow from inbred C57BL/6 mice. Dendritic cells with granulocyte/macrophage-colony stimulating factor were selected	Three different peptides—RVG peptide, a muscle-specific peptide (MSP), and a FLAG epitope—were cloned into Lamp2b	80	Intravenously injected RVG-targeted exosomes delivered glyceraldehyde 3-phosphate dehydrogenase siRNA specifically to neurons, microglia, and oligodendrocytes in the brain, resulting in strong mRNA and protein knockdown of BACE1.	[79]
PD	Dopamine (DA)	Exosome	Blood samples from the orbit venous plexus of Kunming mice containing CD9, CD63, and CD81 marker proteins	-	40–200 (70% in the range of 70–100)	In vitro and in vivo studies demonstrated the successful delivery of DA to the brain using exosomes, including the striatum and substantia nigra. Brain distribution of DA increased >15-fold by using the blood exosomes as a delivery system. DA-loaded exosomes showed much better therapeutic efficacy in 6-OHDA-treated mice and lower systemic toxicity than free DA after intravenous administration.	[80]
PD	Catalase	Exosome	Raw 264.7 macrophages	-	100–200	Exosomes were readily taken up by neuronal cells in vitro. A considerable number of exosomes was detected in 6-OHDA-treated mice brains following intranasal administration, providing significant neuroprotective effects in in vitro and in vivo models of PD.	[81]
PD	Synuclein small interfering RNA	Exosome	Murine dendritic cells harvested from bone marrow	Expressing RVG	100	In normal and in S129D α-syn transgenic mice, the authors detected significantly reduced α-synuclein mRNA and protein levels throughout the brain after treatment with RVG exosomes loaded with siRNA for α-synuclein.	[82]
PD	DNA aptamer targeting α-synuclein aggregates	Exosome	HEK293T cells	Expressing RVG-Lamp2b	-	The aptamer-loaded RVG exosomes significantly reduced the α-synuclein preformed fibril-induced pathological aggregates and rescued synaptic protein (synapsin II and SNAP25) loss and neuronal death. Additionally, intraperitoneal administration of these exosomes into mice treated intra-striatally with α-synuclein preformed fibrils reduced the pathological α-synuclein aggregates and improved motor impairments.	[83]

Alzheimer’s disease (AD), amyloid beta (Aβ), AKT/glycogen synthase kinase-3β (AKT/GSK-3β), beta-secretase (BACE1), blood–brain barrier (BBB), dioleoyl-3-trimethylammonium-propane chloride (DOTAP), 6-hydroxydopamine (6-OHDA), human kidney embryonic cell (HEK293), muscle-specific peptide (MSP), neurofibrillary tangles (NFTs), Parkinson’s disease (PD), rabies virus glycoprotein peptide (RVG), gene associated with brain diseases (SNAP25), α-syn transgenic mice (S129D), synaptosome-associated protein 25 (SNAP25).

**Table 3:** Examples of drugs loaded into solid lipid nanoparticles (SLN) for neurodegenerative and neuropsychiatric disorder treatment.

Target/Disease	Drug(s)	Nanoparticle Type	Composition	Functionalization	Diameter of the Optimized Formulation(s) (nm)	Observations	Ref.
AD	Nicotinamide	SLN	Stearic acid and phospholipon^®^ 90 G (as the oil phase) and sodium taurocholate and ultrapure water (as the water phase)	Polysorbate 80, phosphatidylserine or phosphatidic acid	112, 124, 137	The results of Morris water maze and also histopathological and biochemical tests demonstrated the effectiveness of intraperitoneal injection of phosphatidylserine-functionalized SLNs in improving cognition, preserving the neuronal cells, and reducing tau hyperphosphorylation in a rat model of AD.	[84]
AD	Galantamine hydrobromide	SLN	Compritol (glyceryl behnate), pluronic F-127 (250 mg) as surfactant and Tween 80 as co-surfactant	-	88–221	In vivo assays demonstrated a significant memory restoration capability in cognitive deficit rats in comparison with free drug. The developed carriers offered approximately twice the bioavailability of the free drug.	[85]
AD	Quercetin	SLN	Compritol and Tween 80 as the surfactant	-	159	In all the in vivo behavioral (spatial navigation task, elevated plus maze paradigm, gross behavioral activity) and biochemical (lipid peroxidation, glutathione levels, and nitrite levels) experiments, the rats treated with SLN-encapsulated quercetin showed markedly better memory retention than free quercetin-treated rats.	[86]
AD	Piperine	SLN	Glycerol mono stearate, Epikuron 200, Tween 80, Tween 20	Polysorbate-80 coating	312	Ibotenic acid-stimulated lesions of basalis magnacellularis were developed in albino Wister rats. SLNs containing piperine reduced the SOD values, increased the AChE values, reduced immobility in the forced swimming test, and showed superior results than donepezil. Histopathology studies revealed reduced plaques and tangles.	[87]
AD	Epigallocatechin-3-*O*-gallate (EGCG)	SLN	-	-	50	Nano-EGCG more than doubled the oral bioavailability of EGCG in rats and also was more effective at promoting α-secretase activity in vitro.	[88]
AD	Curcumin	SLN	Soy lecithin and water	Polysorbate 80	135	Young male Lacca mice were treated with AlCl_3_ to induce alterations in the brain histopathology, loss of cognition, and oxidative damage. Behavioral assessments using a Morris water maze test, biochemical measurements (AChE activity, lipid peroxidation, reduced glutathione, superoxide dismutase, catalase, blood lipid profile), and histopathological analysis were performed. Curcumin SLNs completely reversed the induced alterations.	[89]
PD	Apomorphine	SLN	Glyceryl monostearate (GMS) or polyethylene glycol monostearate (PMS)	-	155, 63	6-OHDA-lesioned rats were orally administered with apomorphine. The total number of rotations increased from 20 to 94 and from 20 to 115 when the drug was administered from SLNs containing GMS and PMS, respectively.	[90]
PD	Ropinirole hydrochloride	SLN	Pluronic F68, stearylamine	-	66	Physicochemical characterization and pharmacodynamic studies.	[91]
PD	Naringenin	SLN	Glycerol monostearate, Tween 80, and F68 non-ionic surfactant	-	135	Neuroprotective activity of naringenin–SLN was evaluated using a rotenone-induced PD model in Wistar rats. Results of behavioral observations (in the Rota rod test) and biomarkers (reduced gluthatione, SOD, CAT, lipid peroxidation) showed that naringenin loaded SLN can exert neuroprotective effects.	[92]
Depression/anxiety	Fluoxetine	SLN	Precirol^®^, lauroglycol™, tween80	-	128–158	The intranasal delivery of the optimal lipid nanoparticle formulation reduced both depressive and anxiety-like behaviors in adult CD1 mice (assessment using a mice marble-burying test (MBT) and mice forced swimming test (FST))	[93]

Alzheimer’s disease (AD), acetylcholinesterase (AChE), catalase (CAT), epigallocatechin-3-*O*-gallate (EGCG), mice forced swimming test (FST), Glyceryl monostearate (GMS), 6-hydroxydopamine (6-OHDA), mice marble-burying test (MBT), Parkinson’s disease (PD), polyethylene glycol monostearate (PMS), superoxide dismutase (SOD), solid liquid nanoparticle (SLN).

**Table 4:** Examples of drugs loaded into nanoemulsions (NEs) for neurodegenerative and neuropsychiatric disorder treatment.

Target/Disease	Drug(s)	Nanoparticle Type	Composition	Functionalization	Globule Size of the Optimized Formulation(s) (nm)	Observations	Ref.
AD	Memantine hydrochloride	NE	Labrasol, Tween 20, PEG	-	~11	NE displayed antioxidant activity (FRAP and DPPH), and 98% cell viability in the Neuro 2a cell line. Biodistribution results showed NE uptake in the brains of rats when administered intranasally.	[97]
AD	Donepezil	NE	Labrasol, cetyl pyridinium chloride, glycerol	-	65	Cell viability in the Neuro 2a cell line was 76.3% for the NE and 85% for donepezil solution. In vivo assays showed that rats intranasally administered with the NE showed maximum distribution in the brain and it remained in the target site until 24 h, while no brain uptake was seen in the rats orally administered with the NE. The rats intravenously administered with the NE or intranasally administered with donepezil solution showed only trace amounts of drug distribution in the brain.	[98]
AD	Huperzine A	NE	Isopropylmyristate, Capryol 90, Cremophor EL + Labrasol	Lactoferrin (Lf)	15 (without Lf), 17 (with Lf)	Lf–huperzine A-NE showed better uptake for the hCMEC/D3 cell line than unmodified huperzine A-NE. Similar results were found in in vivo assays.	[99]
AD	Resveratrol	NE	Coconut oil, pluronic and cremophor EL	-	111	In Wistar rats, the brain-targeted efficiency was higher after intranasal administration of resveratrol NE (2 mg/kg) compared with that of resveratrol suspension.	[100]
AD	Curcumin + resveratrol	NE	Labrafac lipophile/cremophor 40, with hyaluronic acid		115	NEs displayed antioxidant activity (DPPH method). Moreover, the integrity of the lining epithelium of the nasal cavity was maintained after repeated administration of hyaluronic-acid-based NEs co-encapsulating curcumin and resveratrol, with no inflammatory cellular infiltration in the lamina propria of the mucosal lining. NEs successfully increased the amount of both polyphenols in the brain of male Albino rats.	[101]
PD	Selegiline	NE	Grape seed oil, Sefsol 218^®^, Tween 80, lauroglycol 90	-	61	Behavior studies (FST, locomotor activity test, catalepsy, muscle coordination test, akinesia test, pole test) were undertaken. Rats were treated with haloperidol to induce PD. Selegiline NE (administered intranasally) showed significant improvement in behavioral activities in comparison to the orally administered free drug.	[102]
PD	Quercetin	NE	Capmul MCM NF and cremophor RH 40	-	~50	In vivo results show that quercetin-loaded NEs potentially reduced α-synuclein aggregation, increased mitochondrial and fat content, and improved the lifespan in transgenic *C. elegans* strain NL5901. Moreover, quercetin NEs significantly downregulated the reactive oxygen species (ROS) levels in wild-type *C. elegans strain* N2 more efficiently than pure quercetin.	[103]
PD	Naringenin	NE	Vitamin E: Capryol 90 (1:1), Tween 80, transcutol-HP, and water	-	39	Behaviors in 6-OHDA-treated rats were successfully reversed after intranasal administration of naringenin NE along with the levodopa. Naringenin NE + levodopa also induced higher levels of GSH and SOD and lower levels of MDA.	[104]
Anxiety	Clove volatile oil (contains eugenol)	NE	Coconut oil, polysaccharides from *Agaricus blazei* Murill mushroom, Tween 80, and water	-	227 to 333	Clove volatile oil (CVO), eugenol, a CVO NE, and an empty NE presented low acute toxicity in zebrafish. The CVO NE reduced the anxious-like behavior of adult zebrafish without affecting their locomotor activity. However, CVO and eugenol displayed anxiolytic activity but reduced animal locomotion similarly to benzodiazepines (diazepam). The observed anxiolytic activity of the CVO, eugenol, and CVO NE is linked to the GABAergic pathway.	[105]
Depression	Fluoxetine	NE	Capmul MCM, labrasol, transcutol-P	-	-	Nasal ciliotoxicity studies were performed to evaluate any potential toxic effects of excipients used in the NE formulation on the nasal mucosa. The blank NE displayed no damage to nasal mucosa showing their potential for intranasal delivery.	[106]
Depression	Quetiapine fumarate	NE	Capmul MCM, Tween 80, transcutol P, PEG, water	-	144	In vivo studies were performed in male Wistar rats after intravenous and intranasal administration of quetiapine pure drug and an NE containing quetiapine. Superiority of the NE for intranasal delivery was observed.	[107]
Depression	Paroxetine	NE	Capmul MCM, Solutol HS 15, and PEG	-	59	Permeation studies revealed increased permeation of the paroxetine NE (2.57-fold) compared to the paroxetine suspension. Behavioral studies (FST and locomotor activity test) in Wistar rats showed that treatment with paroxetine NE (administered intranasally) significantly improved the behavioral activities in comparison to paroxetine suspension (orally administered). The NEs were also effective in counterbalancing oxidative stress.	[108]

Alzheimer’s disease (AD), clove volatile oil (CVO), 2,2-diphenyl-1-picrylhydrazyl (DPPH), Ferric-Reducing Antioxidant Power Assay (FRAP), forced swimming test (FST), gamma-aminobutyric acid (GABA), reduced glutathione (GSH), lactoferrin (Lf), nanoemulsion (NE), 6-hydroxydopamine (6-OHDA), polyethylene glycol (PEG), Parkinson’s disease (PD), reactive oxygen species (ROS), superoxide dismutase (SOD).

**Table 5:** Examples of drugs loaded into polymeric nanoparticles for neurodegenerative and neuropsychiatric disorder treatment.

Target/Disease	Drug(s)	Nanoparticle Type	Composition	Functionalization	Diameter of the Optimized Formulation(s) (nm)	Observations	Ref.
AD	Rivastigmine	Chitosan-based polymeric nanoparticles	Chitosan, tri-polyphosphate pentasodium	Tween 80^®^	154	Compared to both pure rivastigmine and uncoated nanoparticles, Tween 80^®^-coated nanoparticles induced significant reversal of scopolamine-induced effects. Coated nanoparticles also increased the maximum tolerable dose of rivastigmine.	[115]
AD	Rivastigmine	Chitosan-based polymeric nanoparticles	Chitosan, sodium tripolyphosphate	-	164	Intranasal administration showed higher brain targeting of rivastigmine using chitosan nanoparticles compared to other tested formulations.	[116]
AD	Galantamine	Chitosan-based polymeric nanoparticles	Chitosan, sodium tripolyphosphate	Tween 80^®^	48–68	Nasal administration of chitosan nanoparticles exhibited a significant decrease in the AChE protein level and activity in rat brains compared to the oral and nasal free galantamine solutions.	[117]
AD	Tacrine	Chitosan-based polymeric nanoparticles	Chitosan	-	-	The application of tacrine-loaded chitosan nanoparticles selectively increased the tacrine concentration in the brain tissue. Tacrine-loaded non-magnetic and tacrine-loaded magnetic chitosan nanoparticles improved spatial learning and memory after streptozotocin treatment in Wistar rats, with magnetic nanoparticles being the most effective. Tacrine-loaded chitosan nanoparticles increased seladin-1 and reduced APP gene expression.	[118]
AD	Curcumin	Chitosan-based polymeric nanoparticles	Chitosan	Bovine serum albumin (BSA)	144	Curcumin-loaded chitosan/BSA nanoparticles effectively increased drug penetration through the BBB, promoted the activation ofmicroglia, and further accelerated the phagocytosis of the Aβ peptide. They also inhibited the TLR4-MAPK/NF-κB signaling pathway and further downregulated M1 macrophage polarization.	[119]
AD	Piperine	Chitosan-based polymeric nanoparticles	Chitosan, sodium tripolyphosphate, poloxamer 188	-	249	Piperine nanoparticles could significantly improve cognitive functions as efficiently as the standard drug (donepezil injection) with additional advantages of a dual mechanism of action (AChE inhibition and antioxidant effect).	[120]
AD	Thymoquinone	Chitosan-based polymeric nanoparticles	Chitosan, sodium tripolyphosphate	-	172–281	Intranasal thymoquinone-loaded nanoparticles were more effective in brain targeting compared to intravenous and intranasal thymoquinone solutions.	[121]
AD	Memantine	PLGA-based polymeric nanoparticles	Poly(lactic-co-glycolic acid) (PLGA)	PEG	153	Nanoparticles were able to cross the BBB. Behavioral tests in transgenic APPswe/PS1dE9 mice demonstrated that nanoparticles decreased memory impairment in comparison to the free drug solution. Memantine–PEG–PLGA nanoparticles reduced Aβ plaques and the associated inflammation characteristics of AD.	[122]
AD	BACE1-ASshRNA + D-peptide	Dendrigraft poly L-lysines	Dendrigraft poly-l-lysines, α-malemidyl-ω-N-hydroxysuccinimidyl polyethyleneglycol	Two peptides—RVG-29 and D-peptide	97, 110	Downregulation of the key enzyme in Aβ formation was achieved by delivering non-coding RNA plasmid. Simultaneous delivery of the therapeutic gene and peptide into the brain led to a reduction in neurofibrillary tangles. Meanwhile, memory loss rescue in AD mice was also observed.	[7]
AD	Curcuminoids	Cyclodextrin	2-hydroxypropyl-cyclodextrin	-	-	Curcuminoids are rapidly metabolized after intravenous injection into APPSWE/PS1dE9 transgenic mice, and their effect on reducing the plaque load associated with AD may be dependent on the frequency of administration.	[123]
AD	Crocetin	Cyclodextrin	γ-Cyclodextrin	-	-	A water-soluble crocetin-γ-cyclodextrin inclusion complex was nontoxic to normal neuroblastoma cells (N2a cells and SH-SY5Y cells) and AD model cells (7PA2 cells). Furthermore, it showed a stronger ability to downregulate the expression of C-terminus fragments and the level of Aβ in 7PA2 cells compared to the crocetin free drug. Both the inclusion complex and crocetin were able to prevent SH-SY5Y cell death from H_2_O_2_-induced toxicity.	[124]
PD	Rotigotine	PLGA-based polymeric nanoparticles	PLGA	PEG, lactoferrin	122	Qualitative and quantitative cellular uptake studies demonstrated that accumulation of rotigotine was greater when using lactoferrin coating. In addition, intranasal delivery of rotigotine was much more effective with lactoferrin-coated nanoparticles than with uncoated nanoparticles. Rotigotine concentration was higher in the striatum, the primary region affected in PD.	[125]
PD	Rasagiline	Chitosan coated PLGA- polymeric nanoparticles	PLGA	Chitosan	122	Intranasal delivery of mucoadhesive nanocarrier showed significant enhancement of bioavailability in brain, after administration of the rasagiline-chitosan-PLGA-nanoparticles which could be a substantial achievement of direct nose to brain targeting in PD therapy and related brain disorders.	[126]
PD	Puerarin	PLGA-based polymeric nanoparticles	s-PLGA	D-α-tocopherol poly(ethylene glycol)1000 succinate	88	Relative to puerarin alone, encapsulated puerarin exhibited significantly improved cellular internalization, permeation, and neuroprotective effects in zebrafish, rats, and MPTP-treated mice.	[127]
PD	L-DOPA	Cyclodextrin	β-cyclodextrin, 2-hydroxypropyl-β-cyclodextrin	-	-	-	[128]
Anxiety	Buspirone hydrochloride	Chitosan-based polymeric nanoparticles	thiolated chitosan, chitosan, sodium tripolyphosphate, sodium alginate	-	227	The brain concentration achieved after intranasal administration of buspirone/chitosan nanoparticles was higher than that achieved with intravenous or with intranasal administration of free buspirone.	[129]
Anxiety	Quercetin	Polycaprolactone-based polymeric nanoparticles	Polycaprolactone, Span 80, Capryol 90,	Tween 80^®^ or poloxamer 188	228	Behavioral tests demonstrated the superiority of quercetin-loaded polymeric nanocapsules administered intranasally compared to quercetin solution administered both orally and intranasally.	[130]
Depression	Agomelatine	PLGA-based polymeric nanoparticles	PLGA, poloxamer 407	-	116	Pharmacodynamic studies showed a significant reduction in immobility time in forced swimming tests in rats intranasally treated with the formulation, which indicated the antidepressant activity of the formulation.	[131]
Depression	Venlafaxine	PLGA-based polymeric nanoparticles	PLGA	Transferrin or a specific peptide against transferrin receptor	230	In vivo studies showed that non-functionalized nanoparticles reached the brain more efficiently than functionalized ones after intranasal administration. Probably, plain NPs travel via the direct nose-to-brain route whereas functionalized NPs reach the brain via receptor-mediated endocytosis.	[132]
Depression	Venlafaxine	Chitosan-based polymeric nanoparticles	Chitosan, sodium tripolyphosphate	-	168	The higher drug transport efficiency and intranasal direct transport percentage of venlafaxine/chitosan nanoparticles compared to other formulations (non-encapsulated venlafaxine via oral and intranasal route) suggest their better efficacy in the treatment of depression.	[133]
Depression	Venlafaxine	Alginate and chitosan-based polymeric nanoparticles	Alginate, chitosan	-	174	Intranasal administration of venlafaxine-alginate nanoparticles delivered greater amounts of venlafaxine to the brain in comparison to the free drug solution.	[134]
Depression	Desvenlafaxine	PLGA-chitosan polymeric nanoparticles	PLGA, chitosan	-	173	The optimized desvenlafaxine-loaded PLGA/chitosan nanoparticles on intranasal administration significantly reduced the symptoms of depression and enhanced the level of monoamines in the brain in Wistar rats in comparison with orally administered desvenlafaxine. Intranasal delivery of desvenlafaxine PLGA/chitosan nanoparticles also enhanced the pharmacokinetic profile of desvenlafaxine in the brain.	[135]
Depression	Selegiline hydrochloride	Chitosan-based polymeric nanoparticles	Thiolated chitosan	-	215	Forced swimming and tail suspension tests were used to evaluate the antidepressant activity, in which elevated immobility time was found to be reduced upon treatments. Thiolated chitosan nanoparticles seem to be promising candidates for nose-to-brain delivery in the evaluation of antidepressant activity.	[136]
Depression	Sertraline hydrochloride	Cyclodextrin	β-Cyclodextrin	-	-	Physicochemical characterization of the nanoparticles.	[137]
Depression	Fluoxetine hydrochloride	Cyclodextrin	β-Cyclodextrin	-	-	Physicochemical characterization of the nanoparticles.	[137]

Alzheimer’s disease (AD), amyloid beta (Aβ), amyloid precursor protein (APP), , acetylcholinesterase (AChE), blood–brain barrier (BBB), bovine serum albumin (BSA), Cellosaurus cell line CHO 7PA2 (7PA2), 1-methyl-4-phenyl-1,2,3,6-tetrahydropyridine (MPTP), nanoparticles (NPs), Parkinson’s disease (PD), polyethylene glycol (PEG), poly(lactic-co-glycolic acid) (PLGA), rabies virus glycoprotein peptide (RVG), neuroblastoma cell line (SH-SY5Y), Toll-like receptor 4-MAP kinase/ nuclear factor kappa (TLR4-MAPK/NF-κB).

## Data Availability

The data are contained within the article.
